# Alpha-crystallin mutations alter lens metabolites in mouse models of human cataracts

**DOI:** 10.1371/journal.pone.0238081

**Published:** 2020-08-24

**Authors:** Cheryl Frankfater, Stephanie L. Bozeman, Fong-Fu Hsu, Usha P. Andley

**Affiliations:** 1 Mass Spectrometry Resource, Division of Endocrinology, Diabetes, Metabolism, and Lipid Research, Department of Medicine, Washington University School of Medicine, St. Louis, MO, United States of America; 2 Departments of Ophthalmology and Visual Sciences, Washington University School of Medicine, St. Louis, MO, United States of America; University of Colorado Denver School of Medicine, UNITED STATES

## Abstract

Cataracts are a major cause of blindness worldwide and commonly occur in individuals over 70 years old. Cataracts can also appear earlier in life due to genetic mutations. The lens proteins, αA- and αB-crystallins, are chaperone proteins that have important roles maintaining protein solubility to prevent cataract formation. Mutations in the *CRYAA* and *CRYAB* crystallin genes are associated with autosomal dominant early onset human cataracts. Although studies about the proteomic and genomic changes that occur in cataracts have been reported, metabolomics studies are very limited. Here, we directly investigated cataract metabolism using gas-chromatography-mass spectrometry (GC-MS) to analyze the metabolites in adult *Cryaa*-R49C and *Cryab*-R120G knock-in mouse lenses. The most abundant metabolites were *myo*-inositol, L-(+)-lactic acid, cholesterol, phosphate, glycerol phosphate, palmitic and 9-octadecenoic acids, α-D-mannopyranose, and β-D-glucopyranose. *Cryaa*-R49C knock-in mouse lenses had a significant decrease in the number of sugars and minor sterols, which occurred in concert with an increase in lactic acid. Cholesterol composition was unchanged. In contrast, *Cryab*-R120G knock-in lenses exhibited increased total amino acid content including valine, alanine, serine, leucine, isoleucine, glycine, and aspartic acid. Minor sterols, including cholest-7-en-3-ol and glycerol phosphate were decreased. These studies indicate that lenses from *Cryaa*-R49C and *Cryab*-R120G knock-in mice, which are models for human cataracts, have unique amino acid and metabolite profiles.

## Introduction

Cataracts are a major cause of blindness worldwide, and protein aggregation and insolubility are the key sources of lens opacification in human cataractogenesis [1]. Congenital cataracts, which have been linked to crystallin gene mutations, appear early in life and account for approximately 30% of childhood blindness [2–4]. Recently, metabolomics has been used in both clinical and animal studies of several diseases, including some ocular pathologies [5, 6]. However, metabolomic studies specifically focused on cataracts are very limited [7–9]. Early proteomic and RNA-seq studies that investigated the biochemical mechanisms of congenital cataracts suggested that metabolic changes in the lens occur during cataract development. However, these metabolic changes have not been analyzed in detail [7, 10, 11].

Protein quality control is dependent on the proper functioning of chaperone proteins, including α-crystallin [12]. Therefore, it is important to determine how the protein quality control machinery regulates energy metabolism in the lens. As a member of the small heat shock protein family of molecular chaperones, α-crystallin is a major chaperone in the lens that prevents protein aggregation. Importantly, mutant forms of the cryaa and cryab proteins are associated with human cataracts [13, 14], and they may also alter lens metabolism [10, 15]. The arginine-49-to-cysteine mutation (R49C) is associated with congenital cataracts and a loss of chaperone activity of the CRYAA protein [13, 16, 17]. Similarly, the arginine-120-to-glycine mutation is linked to early onset cataracts, a loss of chaperone function of the CRYAB protein, and myopathy [14, 18, 19]. Therefore, to obtain a comprehensive understanding of the metabolic changes that occur downstream of CRYAA and CRYAB human cataract-associated mutations, we analyzed the metabolites in cryaa and cryab knock-in lenses.

The metabolome is the complex array of small-molecule metabolites and metabolic by-products, including carbohydrates, that results from gene expression and protein activity. Importantly, the metabolome can provide new, crucial insights to better understand both healthy and diseased states. Metabolomics relies on analytical platforms, such as proton nuclear magnetic resonance (NMR) spectroscopy and mass spectrometry (MS), including gas chromatography (GC)-MS and liquid chromatography (LC)-MS.

Recent studies have examined the spatial distribution of metabolites in human lenses, the effect of human cataracts on metabolite levels, and the metabolic composition of the rat lens with NMR and LC-MS methods [5, 7–9]. Analyzing the spatial distribution of 34 metabolites in the human lens revealed that most metabolites had a homogenous distribution [8]. Additionally, most metabolites in rat lenses exhibited a gradual decrease with age [9]. Another study found that very low glutathione (GSH) levels were present in the nuclei of cataractous lenses [7]. The concentration of specific lens metabolites can vary significantly under stress, including elevated amino acid levels that were observed in streptozotocin (STZ)-induced and selenite-induced cataracts [20–22]. In vivo models of UV irradiation-induced cataracts also altered the lens metabolite profile [23].

Metabolites reflect the physiological processes that are occurring within the lens and provide important information about pathogenesis. Currently, the precise effects of cryaa and cryab mutations on lens metabolism are unknown; therefore, knock-in mice with *Cryaa* or *Cryab* mutations that are associated with human hereditary cataracts afford a unique opportunity to study cataract lens metabolism. Recently, proteomic and RNA-seq studies with cryaa and cryab mouse models during the early stages of cataract development (postnatal days 2 and 14 lenses) have implicated glycolysis, amino acid biosynthesis, and protein aggregation in cataracts [10, 15].

To determine the functional metabolic ramifications of cryaa and cryab mutations in adult mice, we performed a systematic analysis of the metabolites present in the lenses of adult *Cryaa*-R49C and *Cryab*-R120G knock-in mutant mice and compared them to a wild-type (WT) cohort. We performed GC-MS analysis on hundreds of intermediate metabolites, including lipids, sugars, and amino acids, to identify metabolic predictors of cataracts. Our analytical results can be used to identify interactions between metabolic profiles and lens health that contribute to cataractogenesis.

## Methods

### Animals

All animal procedures were approved by the IACUC at Washington University (St. Louis, MO, USA) and conform to the ARVO Statement for the Use of Animals in Ophthalmic and Vision Research. The Mouse Genetics Core at Washington University was responsible for the mouse care, breeding, and genotyping. WT (wild-type) C57BL/6J and knock-in mice carrying the *Cryaa*-R49C or *Cryab*-R120G mutations were used. Heterozygous (*Cryaa*-R49C-het, *Cryab*-R120G-het) or homozygous (*Cryaa*-R49C-homo, *Cryab*-R120G-homo) mutant knock-in mice were previously generated and studied in our laboratory [19, 24]. The knock-in mutant mice were converted to a C57BL/6J background by speed congenics and identified by strain-specific single nucleotide markers (DartMouse, Lebanon, NH, USA). The metabolite concentration for each mouse lens of each genotype was profiled and the data were averaged by genotype. Adult mice were used for the comparisons between different genotypes. The mice used for the GC-MS analyses were between 113 and 333 days old (mean ± S.D.: 222 ± 73 days). All experiments included at least four lenses per group and were performed in duplicate. Mice ages were as follows: WT, 113–256 days, 160 ± 73, n = 6; *Cryaa*-R49C-het, 273–275 days, 273 ± 1, n = 5; *Cryaa*-R49C-homo, 161–161 days, 161 ± 0, n = 7; *Cryab*-R120G-het, 333–333, 333 ± 0, n = 4; *Cryab*-R120G-homo 192–256 days, 226 ± 29, n = 6.

### Sample preparation

Sample preparation, metabolite quantification, data analysis, and data interpretation were performed at the Washington University School of Medicine Mass Spectrometry Core. The recently described GC-MS methods were used [25–28]. To analyze small metabolites, lenses were dissected from mice according to approved animal protocols. Briefly, the mouse eyes were enucleated, and the lenses were excised and placed in individual microcentrifuge tubes for extraction. The lenses were homogenized with disposable grinders (Axygen, Union City, CA, USA) in 1.5-mL Eppendorf tubes containing 400 μL 100% ethanol. The lens extracts were then centrifuged at 4°C for 30 minutes at 15,000 rpm to pellet the proteins and other insoluble components. Ethanolic supernatants were transferred to a vial insert (Thermo Scientific cat. No. 03-250-630) and dried under nitrogen before derivatizing with 0.2/1/2.8 pyridine/BSTFA 1% TMCS/acetonitrile. The derivatized samples were injected (2 μL with a 1:10 split) and analyzed via an Agilent 7890A gas chromatograph that was coupled to an Agilent 5975C mass spectrometer. The following temperature program was used: 80°C for 2 minutes, followed by a 10°C/min increase until 300°C, and a final hold for 5 minutes.

### Biological data analysis and interpretation

Peak areas were integrated using Chem Station E.02.02.1431 and identified using the National Institute of Standards and Technology (NIST) MS Search 2.3 software and the NIST 2014 (NIST14) and 2017 (NIST17) libraries. Metabolite percentages were calculated as the peak area of the compound/area of the total chromatogram × 100. Compounds were identified using the NIST14 and NIST17 libraries with good (≥ 700) R-matches when compared to the spectra. For a given match, all the peaks in the library spectra had to be present in the experimental spectra of the chromatogram.

Data was imported into Agilent Mass Hunter Version B.07.00, which computationally deconvoluted co-eluting peaks into individual compounds for analysis. The deconvoluted data was then imported into Mass Profiler Professional B.12.6.1 (MPP) to identify metabolite components that were statistically different between genotypes. Within MPP, prior to analysis, the data were normalized, log_2_ transformed, and the median of the control and heterozygous samples were set as the baseline. Because a primary objective was to identify a dose-dependent effect of the mutated gene, one-way analysis of variance comparing the WT, *Cryaa*-R49C-het, and *Cryaa*-R49C-homo genotypes was completed separately from the WT, *Cryab*-R120G-het, and *Cryab*-R120G-homo analysis. *P* values less than or equal to 0.05 were considered statistically significant.

The MPP software provides several methods to filter unreproducible peaks, normalize data, and compare metabolite profiles among groups to identify metabolites that are statistically different between genotypes. To account for differences in lens size and variation in metabolite recovery, we normalized the data using two techniques and compared the results from the two methods. For the 75^th^ percentile technique, the software identified the peak in the 75^th^ percentile of intensity in each chromatogram and made the 75^th^ percentile intensity equivalent across chromatograms. The software then adjusted the intensities of the remaining peaks. We also normalized the peak intensity data using a scalar value of the total ion count of each chromatogram. Both methods yielded the same or very similar results.

In order to clarify the identities of sugar isomers that were difficult to resolve with the NIST libraries alone, we also ran several standards and acquired their retention times and mass spectra (S1 Fig).

## Results

**Fig 1:**
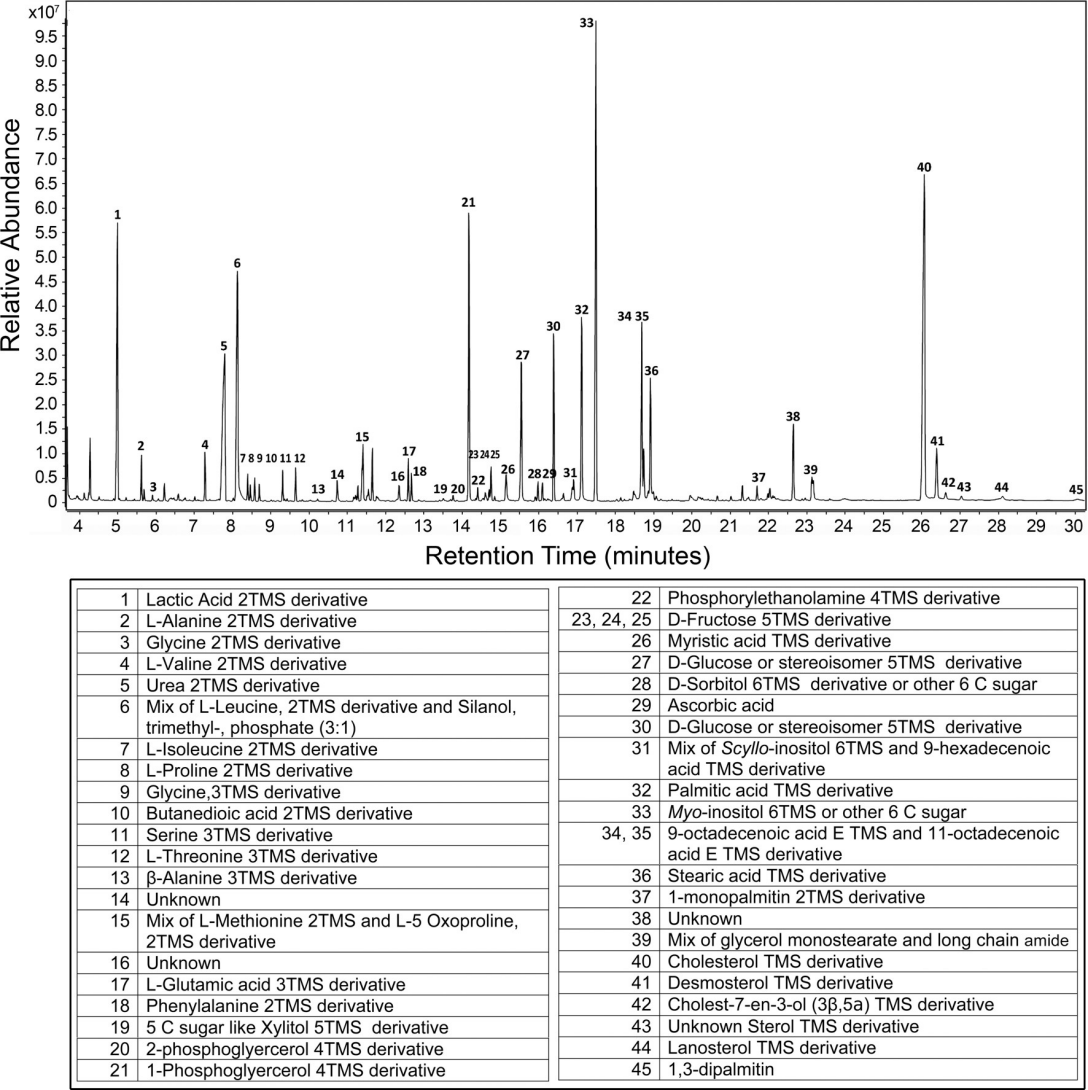
**Representative chromatogram of a lens from a 143-day-old WT mouse showing the elution of amino acids, carbohydrates, and sterols *(top)*.** The peaks in the chromatogram were identified and are shown in the table *(bottom)*.

The lens metabolites that were detectable by GC-MS included amino acids, organic acids, sugars and sugar alcohols, fatty acids, and sterols. One of the most abundant metabolites that was identified was *myo*-inositol (Fig 1). Additionally, TMS derivatives of cholesterol, L-(+)-lactic acid, phosphate, glycerol phosphate, palmitic and 9-octadecenoic acids, and sugars that were identified as β-D-glucopyranose and β-D-galactopyranose, were also prominent on the chromatograms (Fig 1).

Several compounds in the mouse lens extracts could not be identified using the NIST14 and NIST17 libraries or from any standards we analyzed. The mass spectra of these unknown compounds are shown in S2 Fig.

**Fig 2:**
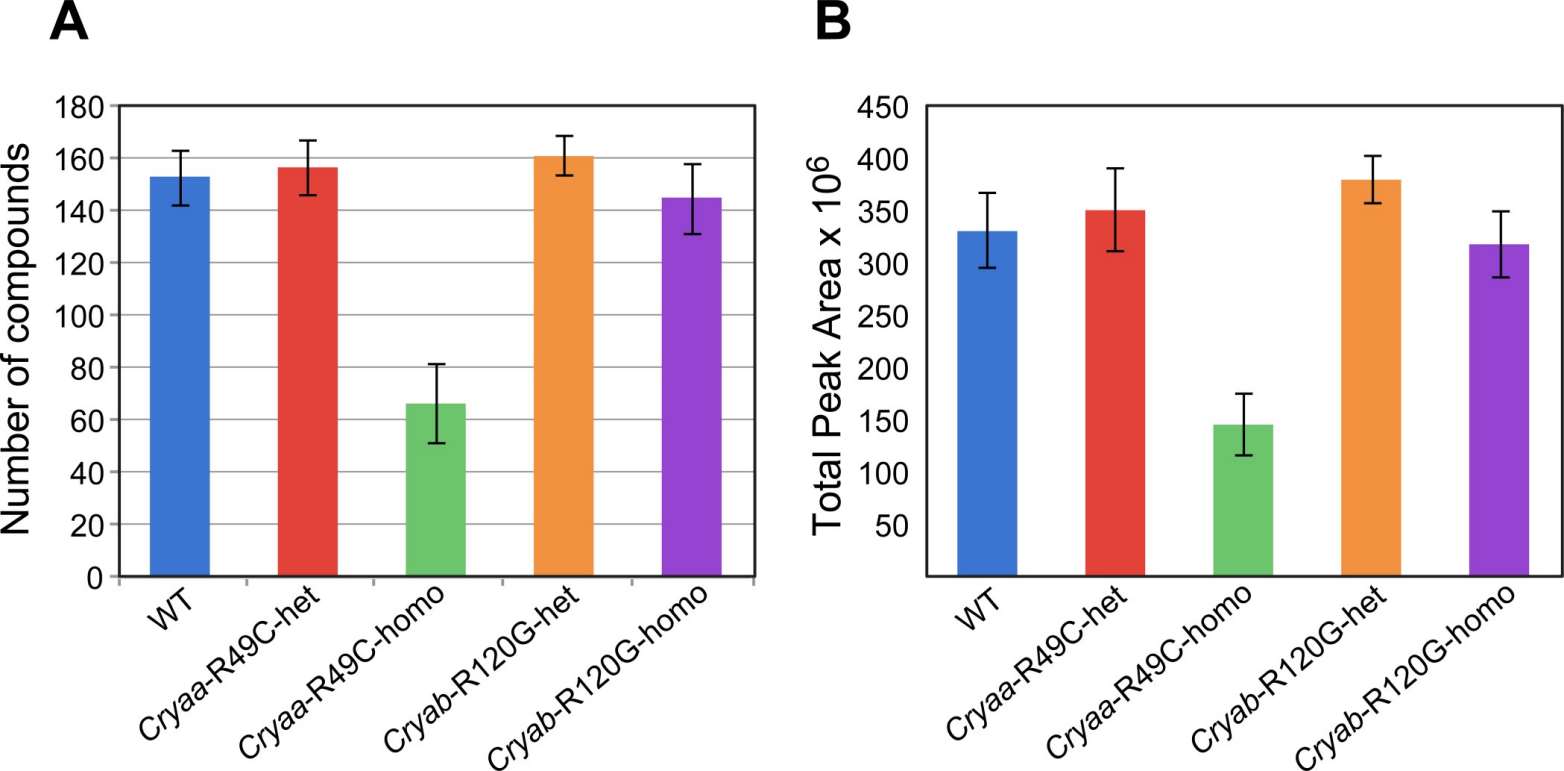
Total number of compounds and average total peak areas in mouse lenses. Data were analyzed using the MPP software. *(A)* The total number of compounds and *(B)* average total peak areas in lenses from WT, *Cryaa*-R49C-het, *Cryaa*-R49C-homo, *Cryab*-R120G-het, and *Cryab*-R120G-homo mice are shown. The mice were 222 ± 73 days old, and the total number of lenses was 34.

**Fig 3:**
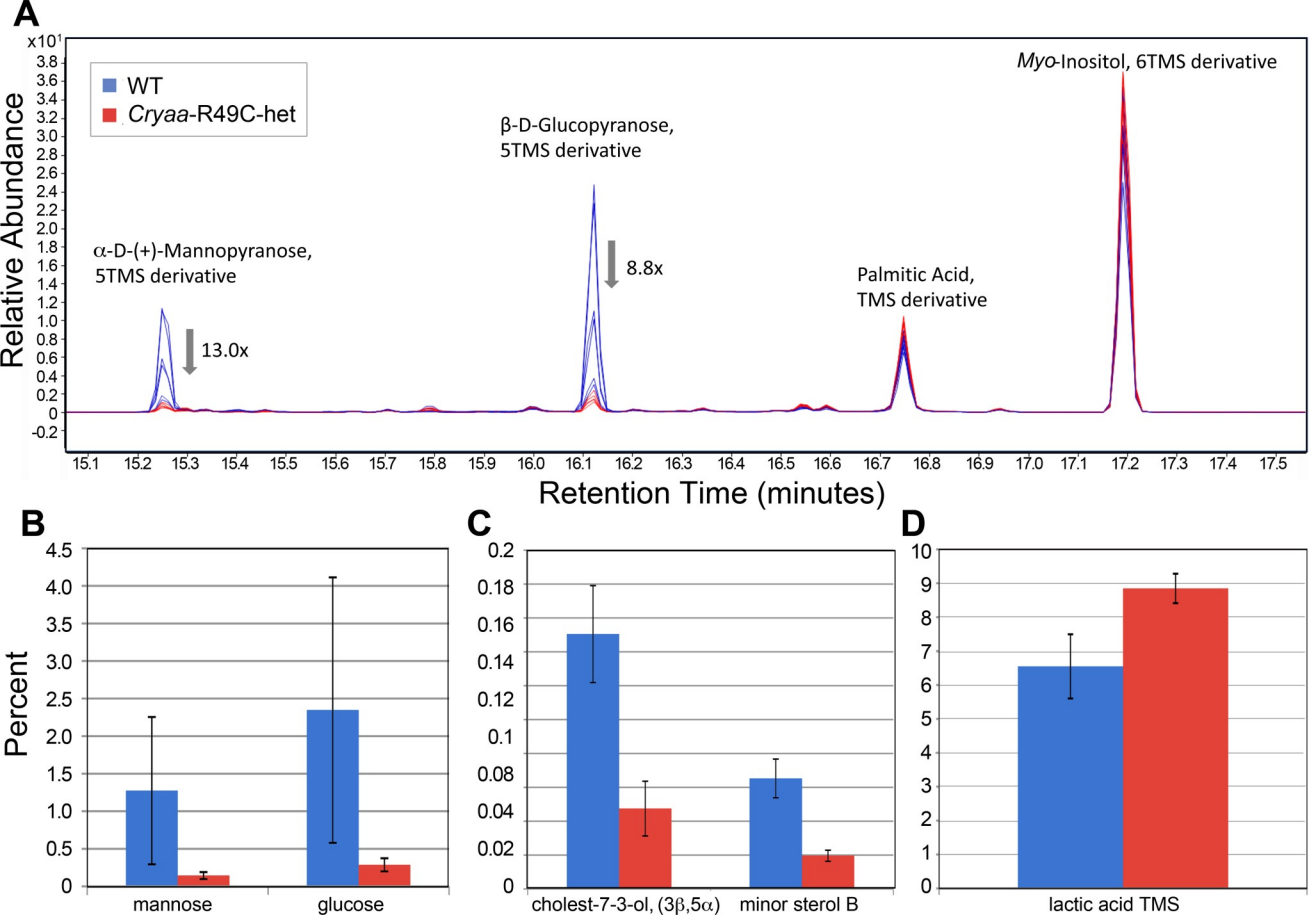
*(A)* Representative chromatograms displaying changes in sugars and sterols in mouse lenses. Sugars *(B)* and sterols *(C)* in lenses from WT and *Cryaa*-R49C-het mice are shown. The sugar and sterol content in the mutant lenses were compared to lenses from WT mice. The match factors were 927 and 946 for α-D-mannopyranose and β-D-glucopyranose, respectively. *(D)* Lactic acid content in *cryaa*-R49C-het mouse lenses were compared to levels in WT mouse lenses. Data are presented as the means ± S.D. (*P* < 0.05).

Because we were most interested in dose-dependent effects of the mutated α-crystallin gene, we first analyzed lenses from the WT, *Cryaa*-R49C-het, and *Cryaa*-R49C-homo mice separate from the WT, *Cryab*-R120G-het, and *Cryab*-R120G-homo mice lenses. The total number of compounds detected was 153 ± 4 (mean ± S.E., n = 6) for WT, 156 ± 5 (n = 5) for *Cryaa*-R49C-het, and 66 ± 7 (n = 7) for *Cryaa*-R49C-homo mice lenses (Fig 2). A small number of metabolites were reduced in *Cryaa*-R49C-het mice. The metabolites at 15.2 and 16.12 minutes were prominently reduced in lenses from *Cryaa*-R49C-het mice and were identified as α-D + mannopyranose and β-D-glucopyranose with match factors of 927 and 946, respectively (Fig 3). Based on our study with the glucose standard, these two isomers were formed with the same retention times upon derivatization of glucose (S1 Fig). Therefore, these two peaks likely represent glucose present in the mouse lenses (S1 Table). The lenses from *Cryaa*-R49C-het mice also exhibited a small (~3 fold) reduction in the relative amounts of minor sterols (Fig 3). The lenses from the *Cryaa*-R49C-het mice had statistically significant decreases in various sugars whose spectra appeared nearly identical but had different retention times (S1 Table). Fig 3D depicts the percentage of lactic acid relative to the other compounds in lenses from WT and *Cryaa*-R49C-het mice. GC-MS analysis revealed an increase in lactic acid in *Cryaa*-R49C-het mouse lenses compared to WT lenses.

Phenotypically, the lenses from *Cryaa*-R49C-homo mice were noticeably smaller and appeared shrunken in size relative to the lenses from WT and *Cryaa*-R49C-het mice [29, 30]. These lenses exhibited dramatic decreases in numerous metabolites, many of which fell below the detection limit, causing them to appear absent or nearly absent on the chromatogram. Therefore, the number of metabolites detected using the MPP software was appreciably reduced (Fig 2). *Myo*-inositol levels in the *Cryaa*-R49C-homo lenses were significantly lower than in the WT lenses (S3 Fig).

**Fig 4:**
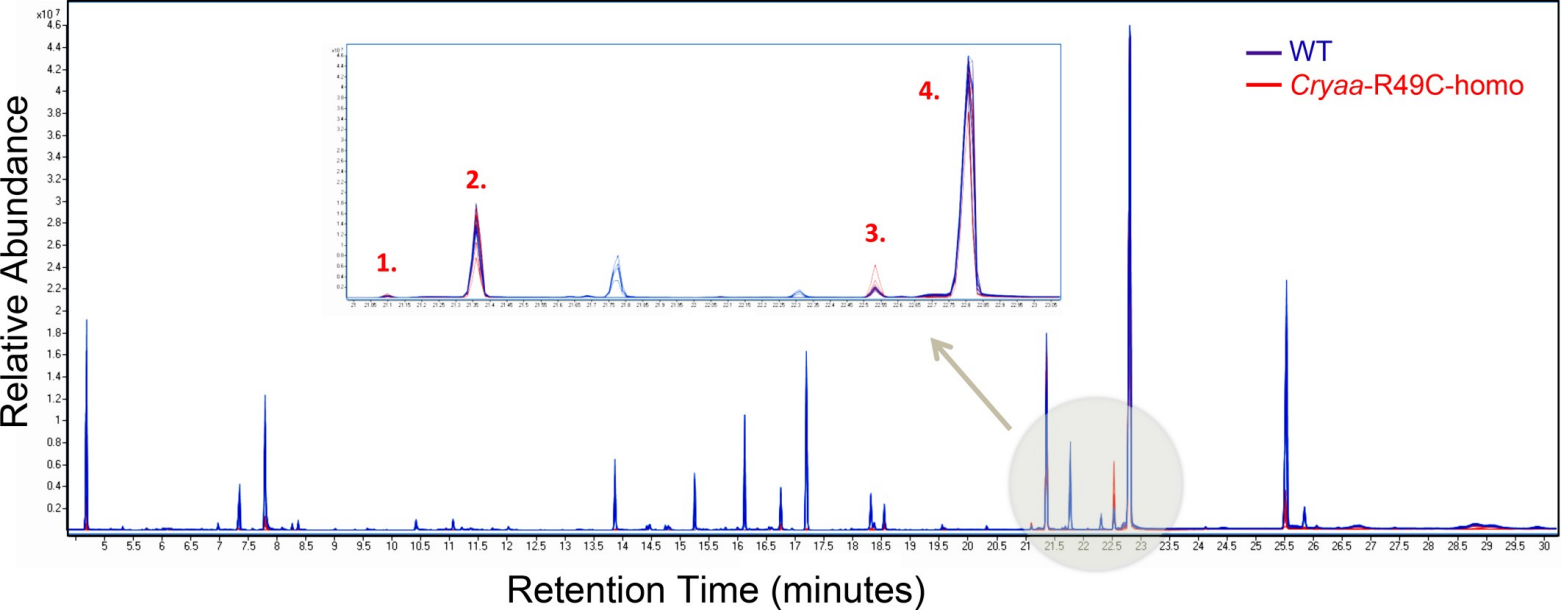
Representative chromatograms of long-chain fatty acids in mouse lenses. Several compounds in the lenses from *Cryaa*-R49C-homo mice were unchanged in abundance compared to lenses from WT mice (*arrow*). The *inset* displays an enlarged view of chromatogram in the *beige circle*. WT, *blue*; *Cryaa*-R49C-homo, *red*. *(1)*, 2-palmitoylglycerol 2 TMS; *(2)*, 1-monopalmitin 2TMS; *(3)*, 2-monostearin 2TMS; *(4)*, glycerol monostearate 2TMS.

Interestingly, in the lenses from *Cryaa*-R49C-homo mice, there was also another subset of compounds that were present in similar amounts to those in the lenses from mice with the WT or *Cryaa*-R49C-het genotypes (Fig 4). These compounds were all classified as lipids and included cholesterol and long carbon chain compounds (2-monostearin, 2-palmitoylglycerol, 9-octadecenoic acid, glycerol monostearate, 1-monopalmitin, 2,3-diydroxypropylicosanoate, stearic acid, butanedioic acid, and palmitic acid). In addition, lactic acid and the 3TMS derivative of phosphate were also prevalent in the chromatograms corresponding to lenses from the *Cryaa*-R49C homozygous mice.

Phosphate was present in the buffer that was used for lens dissection. All lenses were dissected into PBS and gently blotted before being deposited in the ethanol extraction buffer; therefore, the likely origin of the trisilylated phosphate was residual PBS buffer. This peak was also found on many chromatograms in other studies using similar samples, including those derived from cultured cells [31].

**Fig 5:**
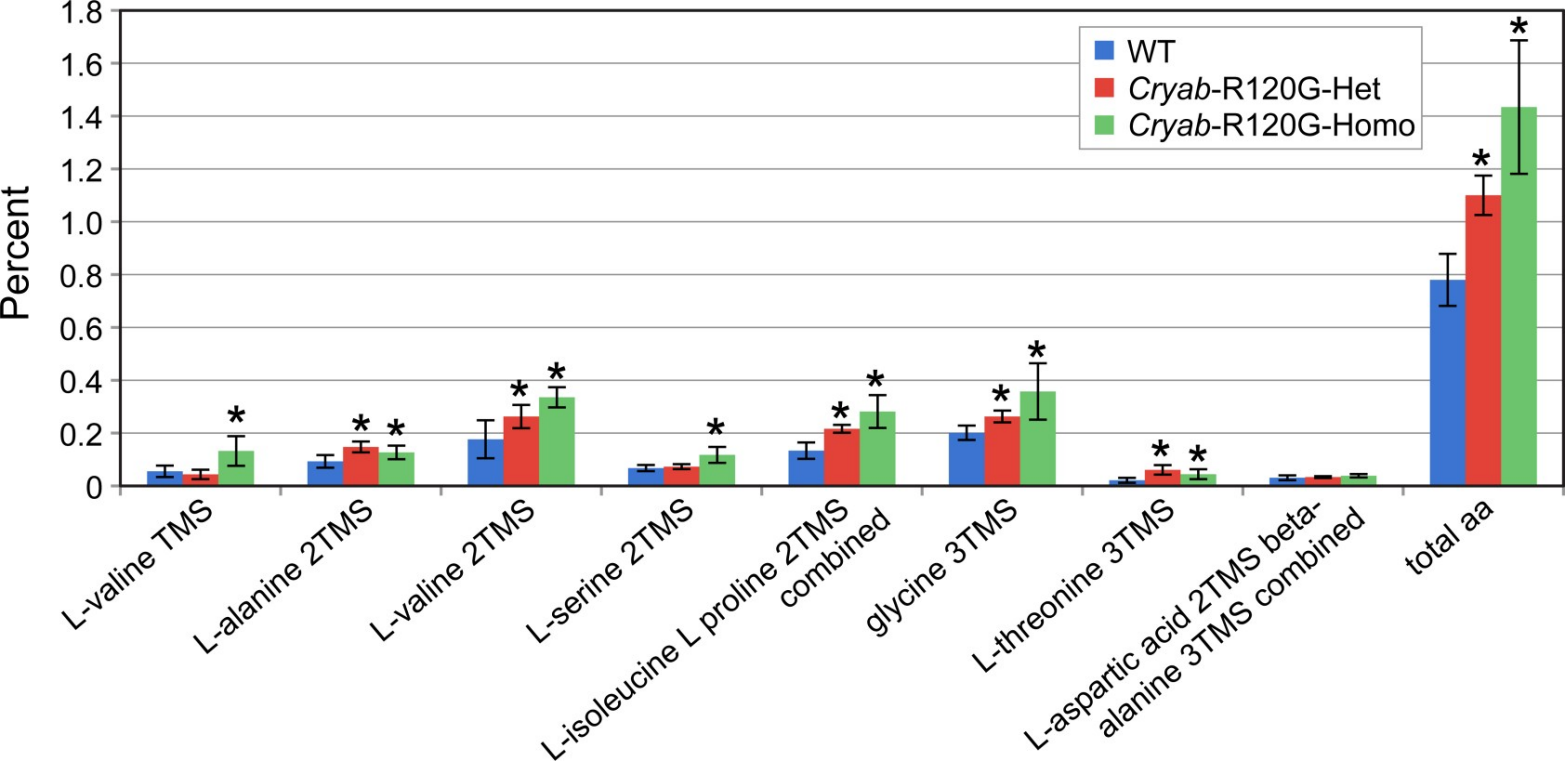
Major and minor sterols in *Cryab*-R120G mouse lenses are compared to WT lenses. Four or six lenses from mice of each genotype were individually analyzed, and the average percentage was determined. Data are presented as the means ± S.D. (*P < 0.05).

We next analyzed lenses from WT, *Cryab*-R120G-het, and *Cryab*-R120G-homo mice. The total number of compounds detected in these lenses was 153 ± 4 (mean ± S.E., n = 6) for WT, 161 ± 4 for *Cryab*-R120G-het (n = 4), and 145 ± 6 (n = 6) for *Cryab*-R120G-homo mice (Fig 2). Relative changes in the percentages of major and minor sterols in lenses from mice with the *Cryab*-R120G-het and *Cryab*-R120G-homo genotypes are shown in Fig 5. Sterols were identified using the NIST14 and NIST17 libraries. However, the sterol at 26.43 minutes (peak 43, Fig 1) was not present in the library. The mass spectra of this and other unidentified peaks is shown in S2 Fig. Changes in the percent of minor lens sterols from mice with the *Cryab*-R120G genotype compared to those in WT mice are displayed in Fig 5.

Lenses from heterozygous mice, which includes both *Cryab*-R120G-het and *Cryaa*-R49C-het, did not appear morphologically different that lenses from WT mice. However, they exhibited quantitative differences in some metabolite levels. There was no appreciable appearance or disappearance of any metabolites. *Myo*-inositol, a major lens metabolite (Fig 1) in *Cryab*-R120G-homo lenses, was present at a lower level than in WT lenses, but this decrease was not statistically significant (S3 Fig).

Two unknown metabolites (retention times 10.42 and 11.75 minutes), minor sterols (retention time 26.4 minutes), cholest-7-en-3-ol (3β, 5 α), and glycerol phosphate decreased in the lenses from *Cryab*-R120G-het and *Cryab*-R120G-homo mice (Fig 5 and S2 Table). The *Cryab*-R120G-homo genotype did not appear to be nearly as severe as the *Cryaa*-R49C-homo genotype because the lenses were not completely shrunken, nor did they exhibit large-scale decreases.

**Fig 6:**
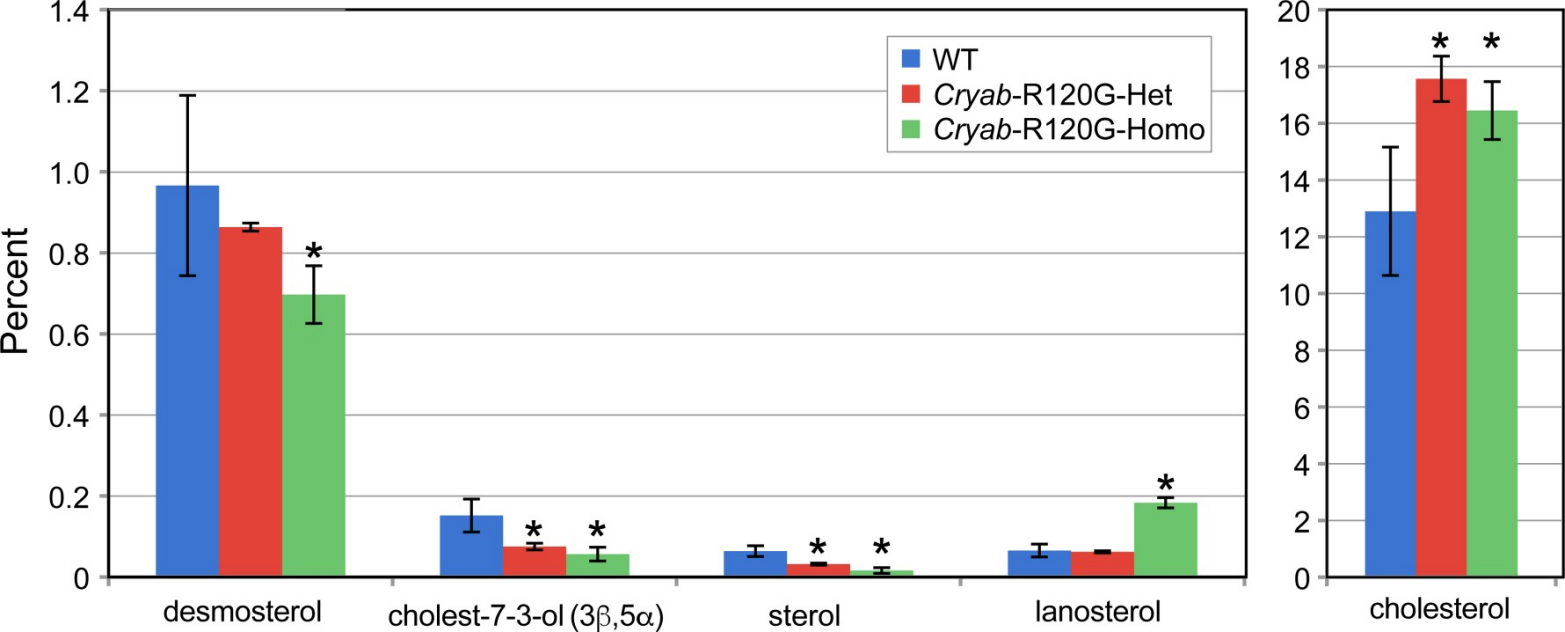
Amino acid content in *Cryab*-R120G mouse lenses are compared to WT mouse lenses. The lenses were first derivatized with TMS, and the amino acids were detected as single- or double-derivatized species. Four or six lenses from mice of each genotype were individually analyzed, and the average percent area of each amino acid was determined. Data are presented as the means ± S.D. (*P < 0.05).

Lenses from *Cryab*-R120G-het and *Cryab*-R120G-homo mice had increased amino acids levels, which occurred in a gene-dose-dependent fashion (Fig 6). The total percentage of amino acids also increased in lenses from *Cryab*-R120G-het and *Cryab*-R120G-homo mice when compared to lenses from WT mice. These changes were statistically significant in lenses from mice with the *Cryab*-R120G-homo genotype. More specifically, L-proline, L-methionine, serine, threonine, L-5-oxoproline (glutamate degradation product), and the branched-chain amino acids L-valine and L-isoleucine increased relative to lenses from WT mice. The L-5-oxoproline peak may be a glutamate degradation product.

We also performed an age-dependent metabolite study with lenses from *Cryab*-R120G-het mice that were 74–143 days (young) and 411–756 days old (aged). We analyzed nearly equal numbers of mice in these two age groups, finding a large difference in metabolite levels between them. Conversely, we showed that the differences in the relative levels of metabolites extracted from WT or cryab mutant lenses in mice between the ages of 100–300 days were exceedingly small and not statistically significant. We measured a statistically significant increase in the percent cholesterol with age, which was accompanied by a decrease in desmosterol content (S4 Fig). In lenses from *Cryab*-R120G-het mice, cholesterol and β-D-glucopyranose emerged as entities that changed proportionally with age. The cholesterol result was more pronounced when compared to β-D-glucopyranose, which may be attributed to the greater cholesterol content in mouse lenses. Some of the lower abundance sterols may also vary with age, but they were present in minor amounts and were not analyzed further.

## Discussion

The in vivo biological consequences of a *Cryaa* or *Cryab* mutation on the lens can be understood by quantitatively measuring the metabolic response of lenses expressing mutant and aggregation-prone forms of cryaa and cryab in knock-in mouse models. Here, we report metabolomic abnormalities in WT and *Cryaa*-R49C and *Cryab*-R120G knock-in mutant lenses using GC-MS as analytical platforms. We used knock-in mouse models that were previously generated in our lab to express mutant cryaa and cryab proteins [19, 24]. The mutant proteins in these models are expressed from birth in every cell; therefore, by studying adult knock-in mice, metabolite changes that occur when a lens grows throughout the lifespan in the presence of mutant cryaa or cryab can be used to better understand inherited human cataracts. This model system can also be used to better understand how genetic perturbations in chaperone proteins affect metabolites.

Several important assumptions within this study require further discussion. In this study, whole lenses were homogenized, and compounds were extracted from the homogenate; therefore, cataract metabolism represents the average of the entire lens. This average does not reveal differences in metabolism of the non-cataractous fractions compared to the cataractous fractions. Due to the very small size of the mouse lens, we did not attempt to dissect the lens to separate the aggregated from non-aggregated fractions. Nevertheless, data for the altered metabolite profiles obtained from heterozygous lenses revealed statistically significant differences between mice with the *Cryaa*-R49C genotype and WT mice in our multivariate analysis. Many metabolites in the extracts from *Cryaa*-R49C-homo lenses, if present, fell below the limits of detection.

Our data demonstrated that carbohydrates and minor sterols decreased in lenses from *Cryaa*-R49C-het mice, which occurred in concert with increased lactic acid content. Because lactic acid is a product of pyruvate reduction, it may be a measure of tricarboxylic acid cycle dysfunction. Collectively, these results suggest an increase in glycolysis and are supported by reduced sugar levels. In addition, we demonstrated that there was an increase in total and specific amino acids and cholesterol in lenses from *Cryab*-R120G-het and *Cryab*-R120G-homo mice. These changes were accompanied by a decrease in desmosterol and other minor sterols in the lenses. Other studies have reported amino acid increases in cataractous lenses [20]. Intriguingly, both mutations affected minor sterol and sugar levels. The lenses from *Cryab*-R12G-het mice had minor increases in monooleoyl glycerol, decreases in 1-octadecanol and desmosterol, and a prominent increase in cholesterol.

The most abundant metabolites that we found in mouse lenses were *myo*-inositol and lactate, which is in agreement with a previous study on human lenses [7]. In the prior study on human lenses with LC-MS, other abundant metabolites were creatine, GSH, glutamate, glucose, and glutamine. The study also showed that metabolite concentration in the cortex and nucleus was similar except for ascorbate, GSH, and NAD, which were lower in the center of the lens than in the cortex. Primary UV filters were also lower in the center of the lens. In human lenses, GSH and ascorbate are in millimolar concentrations, and their levels decrease in cataracts [7].

Another study reported the content of 33 metabolites in rat lenses that were analyzed by GC-MS without preliminary derivatization of the compounds [9]. They reported decreases in several amino acids, including alanine, leucine, and isoleucine, which occurred as age increased from 1 to 14 months. Others have shown that young lenses are more metabolite-rich than old lenses, and alanine, arginine, and glycerophosphate decrease with age [9]. In our study, increased amino acid levels were observed in lenses from the *Cryab*-R120G-het and *Cryab*-R120G-homo mice. These data may indicate elevated degraded protein levels and increased amino acid transport across the cell membrane in mutant lenses. Early work on lens metabolism showed that amino acid oxidation in the lens can be an energy source [32]. In our recent RNA-seq studies with lenses from postnatal *Cryab*-R120G-het mice, we found 5- and 27-fold increases in *Slc6a13* and *Slc1a1* gene expression, respectively [15]. These genes encode the transporters that regulate solute entry into cells, including amino acids. Loss of the amino acid transporter LAT2 *(Slc7a8)* causes a strong imbalance in lens amino acid concentrations and is associated with cataracts in both mouse and humans [33]. In a study that investigated rat lenses and other ocular tissue, 13 lens-specific metabolites were identified by GC-MS [34]. Other studies also report amino acid increases in cataracts [20, 35]. Lens metabolomic profiling has also identified differences in amino acid metabolism between different fish species [36]. Five-oxo-proline, an intermediate of glutamate breakdown (a GSH precursor) was higher in salmon, the species with increased cataract severity and prevalence when compared to trout, the other fish studied. The increase in the branched-chain amino acids leucine, isoleucine, and valine that are traditionally associated with oxidative metabolism and energy production in muscle tissue have also been identified as significant predictors of diabetes. Importantly, the breakdown of branched-chain amino acids may be a source of acetyl coenzyme A in lens epithelial cells, which suggests that branched-chain amino acids may induce oxidative damage and increase the risk of cataract development. Our data demonstrating that amino acid levels increase in cataracts caused by the *Cryab*-R120G mutation could have great potential medical implications.

Glucose is the primary energy substrate for the lens. Several sugars were decreased in the lenses from *Cryaa*-R49C-het and *Cryaa*-R49C-homo mice, which is in agreement with our previous RNA-seq studies that showed decreased *Slc31a1* (-6.5 fold), *Slc46a3* (-4.4-fold), *Slc20a2* (-3.77 fold), and *Slc7a13* (-89 fold). However, *Slc7a3* increased 21- fold in the previous study [15].

Anaerobic glycolysis has an important role in the lens and lactic acid increases during this process. We found increased lactic acid levels in lenses from *Cryaa*-R49C-het mice when compared to lenses from WT mice. We also found decreased phosphoglycerol in lenses from *cryaa*-R49C-het mice when compared to lenses from WT mice.

*Myo*-inositol helps to regulate important cellular functions including glucose homeostasis. Notably, low levels of *myo*-inositol are associated with diabetes and disrupted cell signaling pathways, including with inositol triphosphate (IP3) and phosphatidylinositol phosphate lipid (PIP2/PIP3) signaling. *Myo*-inositol also contributes to cell growth and survival. Lens health is also dependent on *myo*-inositol. Lenses have Na^+^-dependent *myo*-inositol transport and high levels of *myo*-inositol cause osmotic stress that contributes to cataract formation. Low levels of *myo*-inositol occur in diabetic rats with cataracts, but can be resolved with diet modifications [37]. Although an osmotic effect can explain the physical changes in the lens that lead to cataract formation, sorbitol accumulation in other tissues and the ensuing diabetic complications are associated with *myo-*inositol depletion and disrupted Na^+^/K^+^-ATPase activity [38, 39]. Our data demonstrating reduced levels of *myo*-inositol in *Cryaa*-R49C-homo lenses may indicate its role in the formation of cataracts caused by this mutation.

Current lens metabolomic studies provide the foundation for future, detailed analyses and can even be extended to targeted metabolomics. Instruments that are suitable for metabolic flux analysis with ^13^C or ^2^H tracers and targeted metabolomics would enhance these studies. MS has been used to quantify metabolic fluxes in glycolysis, the TCA cycle, pentose phosphate pathways, amino acid metabolism, nucleotide metabolism, and lipid metabolism. These studies provide insight about the effects of poor protein quality control due genetic mutations in a major crystallin gene and the downstream effects on glucose metabolism. We showed that after expressing *Cryab*-R120G crystallin in the lens, glycolytic intermediates and amino acids were altered and glycolytic flux was enhanced. In addition, sugar levels were reduced, and lactic acid production was enhanced in *Cryaa*-R49C expressing heterozygous lenses. These metabolic fluctuations reflect changes in lens biology caused by mutant cryaa or cryab protein expression.

## Supporting information

S1 Fig GC-MS analysis of standard compounds (related to Fig 1). Mass spectral analysis of D-glucose, D-fructose, and *myo*-inositol by GC-MS are shown. *(A)* D-glucose yielded two peaks at retention times 15.6 and 16.4 minutes, close to peaks 27 and 30 in the WT mouse lens extracts shown in Fig 1. These peaks were identified as 5TMS derivatives, β-D-(+)-mannopyranose and β-D-glucopyranose, respectively. *(B)* The retention times for D-fructose peaks at 14.70, 14.78 and 14.86 minutes were close to peaks 23, 24, and 25 in the WT mouse lens extracts shown in Fig 1. *(C) Myo*-inositol, 6TMS derivative, at retention time 17.6 minutes was close to peak 33 in the WT mouse lens extracts shown in Fig 1.
(TIF)

S2 Fig Mass spectra of unknown compounds detected in mouse lens extracts (related to Fig 1). The mass spectra of four compounds present in mouse lens extracts that could not be definitively identified using NIST14 and NIST17 library searches are shown. Data from a WT mouse lens are shown in Fig 1. *(A)* Peak 14 (retention time 10.73 minutes). *(B)* Peak 16 (retention time 12.34 minutes). *(C)* Peak 38 (retention time 22.64 minutes). *(D)* Peak 43 (retention time 27.01–27.04 minutes).
(TIF)

S3 Fig *myo*-inositol in mouse lenses (related to Fig 3). Graphs showing percentages of *myo*-inositol levels in WT, *Cryaa*-R49C-het, *Cryaa*-R49C-homo, *Cryab*-R120G-het, and *Cryab*-R120G-homo mouse lenses analyzed by GC-MS.
(DOCX)

S4 Fig Age-related changes in cholesterol and desmosterol percentages in lenses from *Cryab*-R210G-het mice. GC-MS analysis reveals a cholesterol increase in *Cryab*-R120G-het mouse lenses with age *(A)*. In contrast, the percentage of desmosterol decreased with age *(B)*. The percentage of β-D-glucopyranose also decreased *(C)*.
(TIF)

S1 Table Change in abundance of metabolites in *Cryaa*-R49C-het and *Cryaa*-R49C-homo knockin mouse lenses as compared with WT lenses*. (DOCX)

S2 Table Change in abundance of metabolites in *Cryab*-R120G-het and *Cryab*-R120G-homo knockin mouse lenses as compared with WT lenses. (DOCX)
