# Correction: Alpha-crystallin mutations alter lens metabolites in mouse models of human cataracts

**DOI:** 10.1371/journal.pone.0242951

**Published:** 2020-11-20

**Authors:** The PLOS ONE Staff

The images for Figs 5 and 6 are incorrectly switched. The image that appears as Fig 5 should be Fig 6, and the image that appears as Fig 6 should be Fig 5. The figure captions appear in the correct order. Please see the correct Figs 5 and 6 here.

The publisher apologizes for the error.

**Fig 5:**
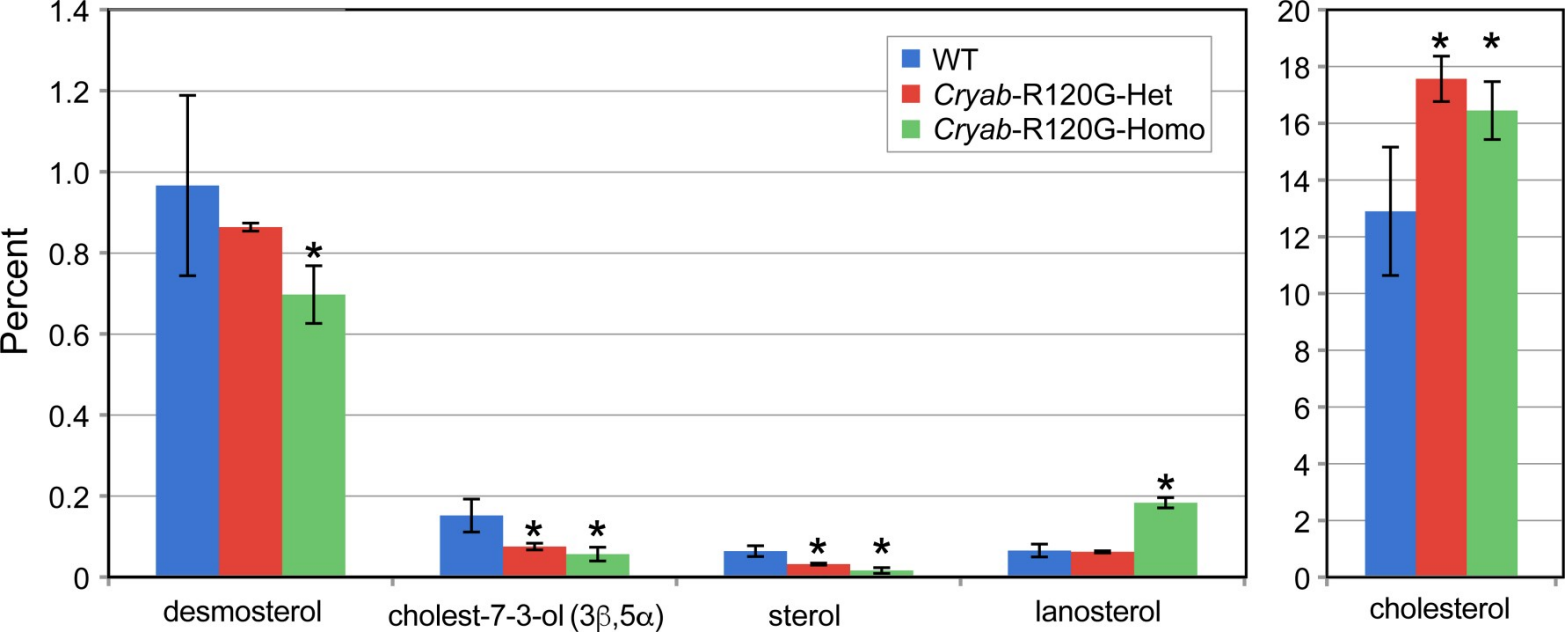
Major and minor sterols in *Cryab*-R120G mouse lenses are compared to WT lenses. Four or six lenses from mice of each genotype were individually analyzed, and the average percentage was determined. Data are presented as the means ± S.D. (*P < 0.05).

**Fig 6:**
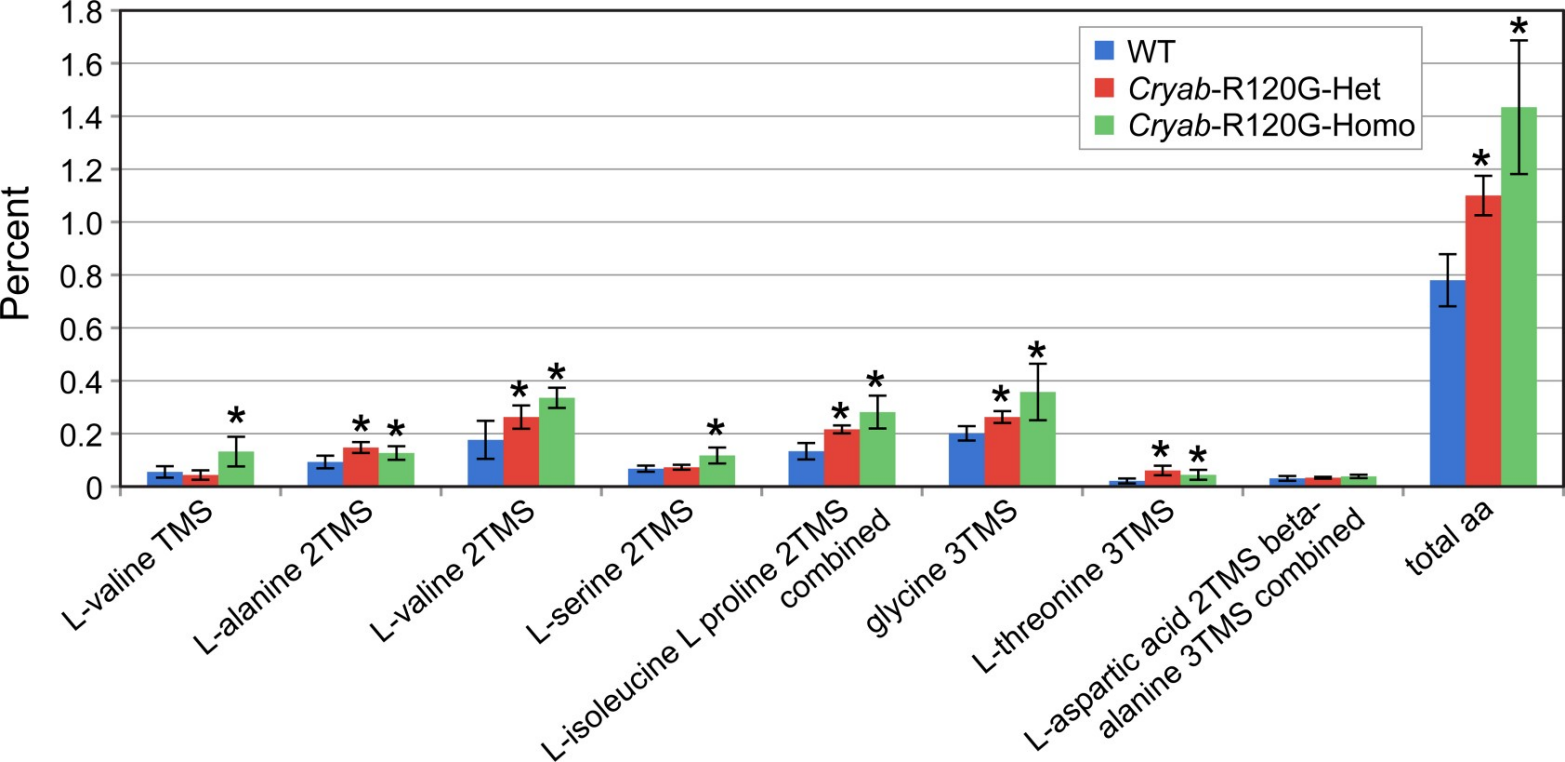
Amino acid content in *Cryab*-R120G mouse lenses are compared to WT mouse lenses. The lenses were first derivatized with TMS, and the amino acids were detected as single- or double-derivatized species. Four or six lenses from mice of each genotype were individually analyzed, and the average percent area of each amino acid was determined. Data are presented as the means ± S.D. (*P < 0.05).
