# Supplementary figures and images for: Alpha-crystallin mutations alter lens metabolites in mouse models of human cataracts
Source: PLoS One. 2020 Aug 24;15(8):e0238081. doi: 10.1371/journal.pone.0238081 (PMC7446835; doi:10.1371/journal.pone.0238081)

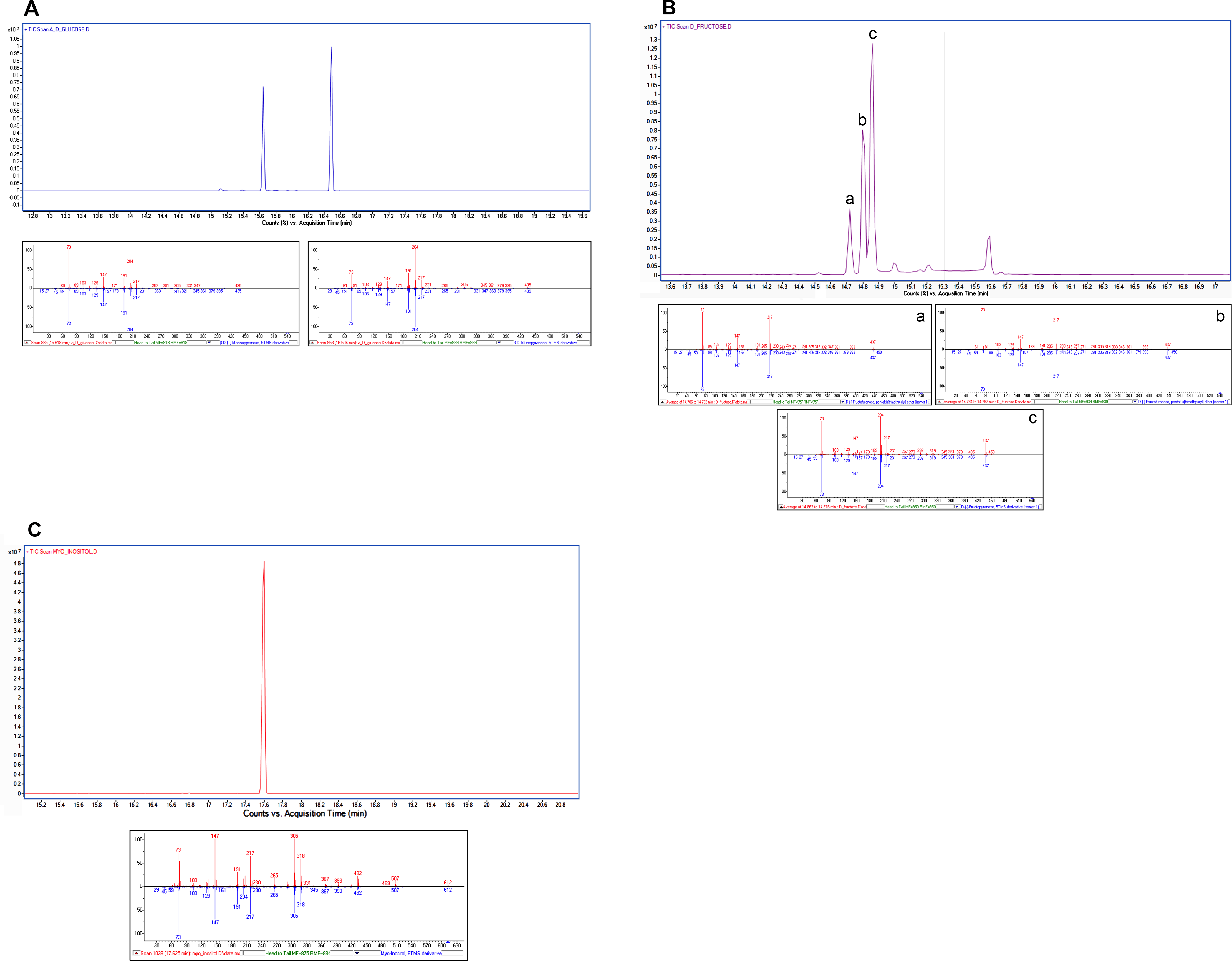

Supplement: S1 Fig — Mass spectral analysis of D-glucose, D-fructose, and myo-inositol by GC-MS are shown. (A) D-glucose yielded two peaks at retention times 15.6 and 16.4 minutes, close to peaks 27 and 30 in the WT mouse lens extracts shown in Fig 1. These peaks were identified as 5TMS derivatives, β-D-(+)-mannopyranose and β-D-glucopyranose, respectively. (B) The retention times for D-fructose peaks at 14.70, 14.78 and 14.86 minutes were close to peaks 23, 24, and 25 in the WT mouse lens extracts shown in Fig 1. (C) Myo-inositol, 6TMS derivative, at retention time 17.6 minutes was close to peak 33 in the WT mouse lens extracts shown in Fig 1. (TIF) [file pone.0238081.s001.tif]

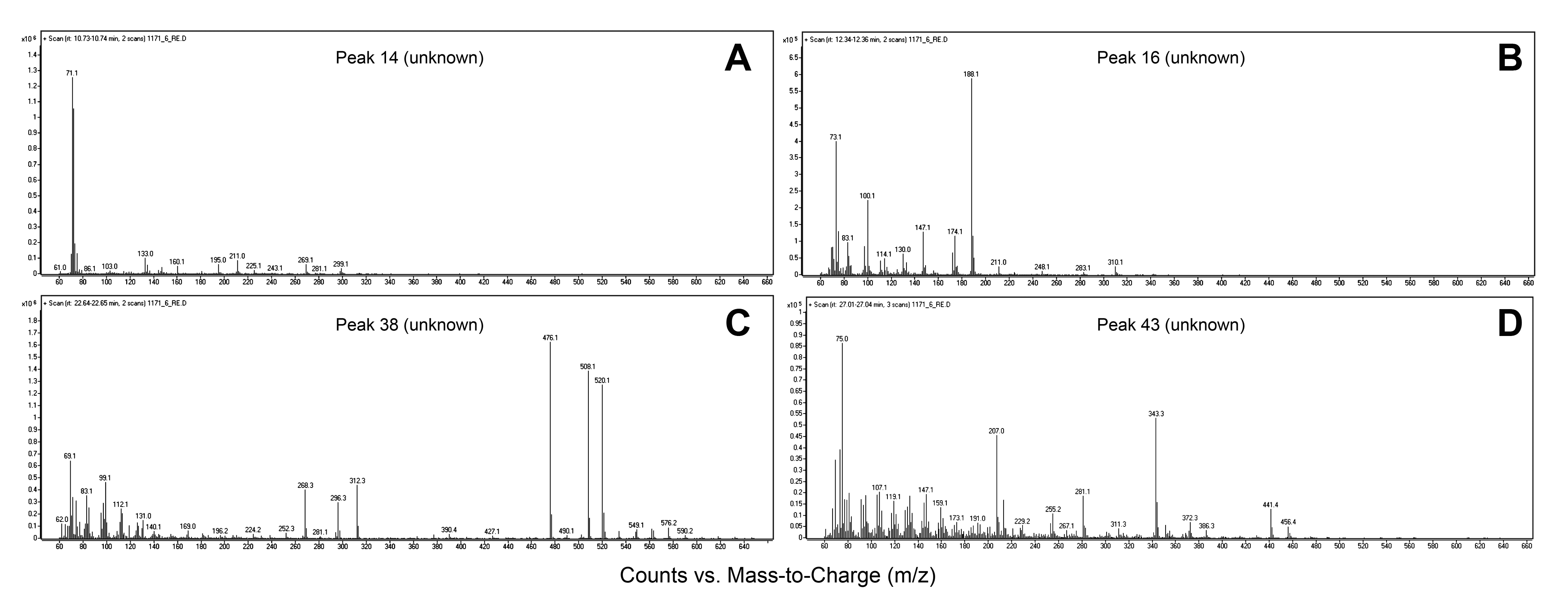

Supplement: S2 Fig — The mass spectra of four compounds present in mouse lens extracts that could not be definitively identified using NIST14 and NIST17 library searches are shown. Data from a WT mouse lens are shown in Fig 1. (A) Peak 14 (retention time 10.73 minutes). (B) Peak 16 (retention time 12.34 minutes). (C) Peak 38 (retention time 22.64 minutes). (D) Peak 43 (retention time 27.01–27.04 minutes). (TIF) [file pone.0238081.s002.tif]

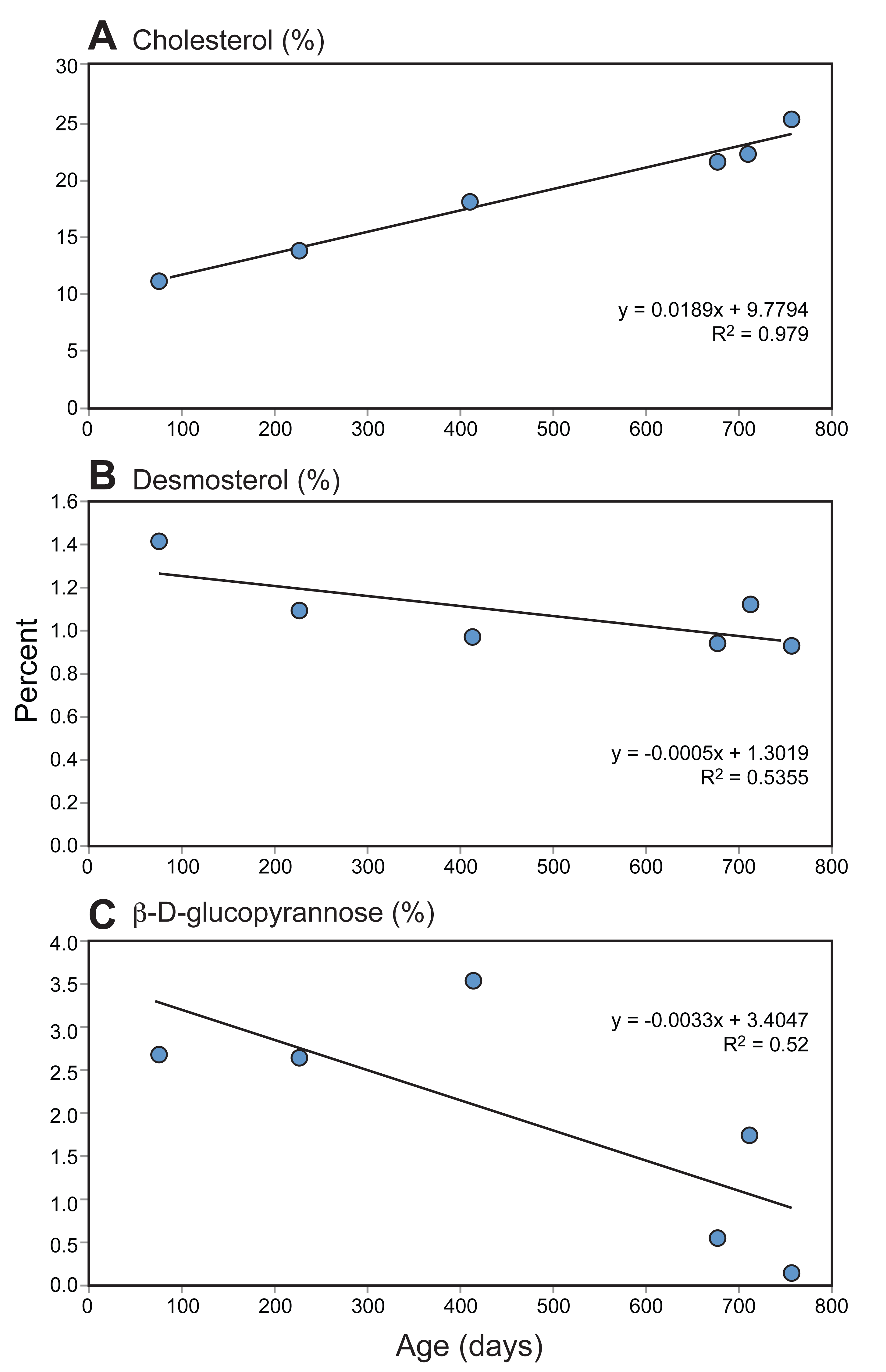

Supplement: S4 Fig — GC-MS analysis reveals a cholesterol increase in Cryab-R120G-het mouse lenses with age (A). In contrast, the percentage of desmosterol decreased with age (B). The percentage of β-D-glucopyranose also decreased (C). (TIF) [file pone.0238081.s004.tif]
