# Supplementary material for: Alpha-crystallin mutations alter lens metabolites in mouse models of human cataracts
Source: PLoS One. 2020 Aug 24;15(8):e0238081. doi: 10.1371/journal.pone.0238081 (PMC7446835; doi:10.1371/journal.pone.0238081)
Supplement: S3 Fig — Graphs showing percentages of myo-inositol levels in WT, Cryaa-R49C-het, Cryaa-R49C-homo, Cryab-R120G-het, and Cryab-R120G-homo mouse lenses analyzed by GC-MS. (DOCX) [file pone.0238081.s003.docx]

**Supplementary Fig. S3**
