# Supplementary material for: Alpha-crystallin mutations alter lens metabolites in mouse models of human cataracts
Source: PLoS One. 2020 Aug 24;15(8):e0238081. doi: 10.1371/journal.pone.0238081 (PMC7446835; doi:10.1371/journal.pone.0238081)
Supplement: S1 Table — (DOCX) [file pone.0238081.s005.docx]

**Table S1.** Change in abundance of metabolites in *Cryaa*-R49C-het and *Cryaa*-R49C-homo knockin mouse lenses as compared with WT lenses*.

|  |  |  | **Fold changes in abundance of metabolites in *Cryaa*-R49 knockin mouse lenses** | | |  |  |
| --- | --- | --- | --- | --- | --- | --- | --- |
| **Compound** | **Match Factor** | **p (Corr)** | ***Cryaa*-R49C-homo vs. *cryaa*-R49-het** | ***Cryaa*-R49C-het vs. WT** | ***Cryaa*-R49C-homo vs. WT** | **Retention Time (min)** | **Mass (Da)** |
| Cholest-7-en-3-ol, (3.beta.,5.alpha.)-, TMS | 882 | 6.99E-05 | -1.71 | -2.74 | -4.68 | 26.06 | 458 |
| unknown |  | 3.53E-08 | -3.67 | -1.74 | -6.39 | 12.02 | 73 |
| D-(-)-Fructopyranose, 5TMS (isomer 1) | 908 | 2.25E-13 | -6.28 | -1.40 | -8.82 | 14.48 | 204 |
| L-Valine, TMS | 912 | 2.38E-05 | -1.85 | -1.28 | -2.37 | 5.12 | 72 |
| L-Leucine, TMS | 864 | 8.27E-07 | -2.46 | -1.25 | -3.07 | 6.12 | 86 |
| Heptadecanoic acid, glycerine-(1)-monoester, bis-O-TMS | 775 | 3.73E-07 | 14621.74 | -1.25 | 11738.02 | 22.08 | 385 |
| Benzene, (1-methyldodecyl)- | 841 | 1.40E-04 | 2535.30 | -1.20 | 2116.86 | 15.46 | 105 |
| 2-Monostearin, 2TMS | 872 | 1.87E-25 | 112075.90 | -1.16 | 96224.16 | 22.53 | 129 |
| 2,3-Dihydroxypropyl icosanoate, 2TMS | 792 | 4.25E-23 | 34883.74 | 1.01 | 35330.59 | 24.13 | 427 |
| unknown |  | 1.56E-07 | -2.24 | 1.04 | -2.16 | 5.91 | 98 |
| 9-Octadecenamide, (Z)- | 834 | 1.36E-05 | 2785.78 | 1.06 | 2939.85 | 19.55 | 72 |
| 2-Palmitoylglycerol, 2TMS | 824 | 1.04E-23 | 62333.83 | 1.07 | 66731.98 | 21.10 | 129 |
| 1-Monomyristin, 2TMS | 733 | 1.73E-04 | 1827.03 | 1.08 | 1968.75 | 19.83 | 343 |
| Tris(trimethylsilyl)carbamate | 632 | 1.15E-24 | 25011.90 | 1.14 | 28450.90 | 4.26 | 73 |
| unknown |  | 2.24E-04 | -1.86 | 1.14 | -1.63 | 7.09 | 112 |
| DL-Phenylalanine, TMS | 915 | 6.67E-05 | -2.62 | 1.15 | -2.29 | 11.40 | 120 |
| unknown |  | 2.64E-09 | -6.16 | 1.15 | -5.37 | 11.21 | 84 |
| Scyllo-Inositol, 6TMS | 704 | 8.03E-08 | -2.61 | 1.16 | -2.24 | 16.21 | 73 |
| 1-Monopalmitin, 2TMS | 941 | 1.65E-25 | 60488.28 | 1.17 | 70840.82 | 21.36 | 371 |
| Silanol, trimethyl-, phosphate (3:1) | 959 | 3.23E-18 | 4435.31 | 1.18 | 5242.92 | 7.79 | 299 |
| Stearic acid, TMS | 927 | 1.65E-25 | 21666.73 | 1.20 | 26019.90 | 18.55 | 117 |
| unknown |  | 2.23E-05 | -3.44 | 1.20 | -2.86 | 11.75 | 73 |
| β-D-Galactofuranose, 1,2,3,5,6-pentakis-O-(TMS)- | 722 | 7.41E-09 | -3.39 | 1.21 | -2.81 | 16.94 | 73 |
| L-Threonic acid, tris(TMS) ether, trimethylsilyl ester | 868 | 2.30E-08 | -3.78 | 1.21 | -3.13 | 11.37 | 73 |
| unknown |  | 1.28E-04 | 1711.53 | 1.22 | 2084.02 | 7.44 | 179 |
| Oxalic acid 2TMS | 885 | 0.001042 | 704.19 | 1.24 | 876.64 | 5.73 | 73 |
| D-Allofuranose, pentakis(trimethylsilyl) ether | 755 | 5.74E-12 | -4.90 | 1.26 | -3.89 | 16.34 | 217 |
| Glycerol monostearate, 2TMS | 944 | 1.51E-22 | 50331.14 | 1.30 | 65192.24 | 22.81 | 399 |
| Palmitic Acid, TMS | 941 | 2.46E-24 | 8820.65 | 1.31 | 11522.44 | 16.75 | 117 |
| unknown |  | 5.12E-16 | -8.08 | 1.34 | -6.04 | 21.69 | 73 |
| Cholesterol, TMS | 936 | 3.33E-16 | 3856.57 | 1.38 | 5324.68 | 25.53 | 129 |
| Butanedioic acid, 2TMS | 918 | 1.97E-20 | 7456.00 | 1.42 | 10620.56 | 8.37 | 147 |
| unknown |  | 2.49E-04 | -3.02 | 1.43 | -2.11 | 7.93 | 181 |
| unknown |  | 0.003826 | -2.37 | 1.46 | -1.62 | 6.70 | 147 |
| Pyroglutamic acid, TMS | 821 | 1.89E-04 | -2.09 | 1.51 | -1.39 | 10.80 | 84 |
| Palmitelaidic acid, TMS | 840 | 7.38E-14 | -9.30 | 1.52 | -6.10 | 16.55 | 117 |
| 9-Octadecenoic acid, (E)-, TMS | 926 | 5.26E-04 | 304.64 | 1.57 | 477.70 | 18.31 | 73 |
| L-Alanine, 2TMS | 922 | 2.89E-12 | -5.69 | 1.57 | -3.62 | 5.32 | 116 |
| Lactic Acid, 2TMS | 955 | 7.38E-14 | 1960.23 | 1.57 | 3084.22 | 4.69 | 147 |
| L-Isoleucine, 2TMS + L-Threonine, 2TMS | 739 | 9.46E-12 | -5.47 | 1.58 | -3.46 | 8.09 | 73 |
| L-Valine, 2TMS | 896 | 4.52E-16 | -11.05 | 1.61 | -6.86 | 6.98 | 144 |
| 1-Monooleoylglycerol, 2TMS | 764 | 1.37E-07 | -2.92 | 1.64 | -1.78 | 22.61 | 73 |
| unknown |  | 1.72E-16 | -28.29 | 1.65 | -17.13 | 10.42 | 71 |
| Lanosterol, TMS | 742 | 2.86E-06 | -2.38 | 1.67 | -1.42 | 27.41 | 69 |
| Silanol, trimethyl-, phosphate (3:1) | 903 | 0.001757 | 655.43 | 1.70 | 1111.86 | 7.82 | 205 |

*The *Cryaa*-R49-homo lenses had a greatly reduced number of metabolites as compared with *Cryaa*-R49-het and WT lenses. After normalization, this resulted in high fold change values for the remaining peaks. MPP generates a table of statistically significant fold-changes based on the relative proportions of each metabolite within a genotype. Some of the positive fold changes in the *Cryaa*-R49-homo lenses appear enormous. These compounds did not increase in quantity; rather because the majority of the other small metabolites diminish to baseline levels, the remaining compounds represent a much larger proportion of the detectable metabolites in that particular genotype.
