# Supplementary material for: Alpha-crystallin mutations alter lens metabolites in mouse models of human cataracts
Source: PLoS One. 2020 Aug 24;15(8):e0238081. doi: 10.1371/journal.pone.0238081 (PMC7446835; doi:10.1371/journal.pone.0238081)
Supplement: S2 Table — (DOCX) [file pone.0238081.s006.docx]

**Table S2.** Change in abundance of metabolites in *Cryab*-R120G-het and *Cryab*-R120G-homo knockin mouse lenses as compared with WT lenses.

|  |  |  | **Fold changes in abundance of metabolites in *Cryab*-R120G knock-in mouse lenses** | | |  |  |
| --- | --- | --- | --- | --- | --- | --- | --- |
| **Compound** | **Match Factor** | **p (Corr)** | ***Cryab*-R120G-homo vs. *Cryab*-R120G-het** | ***Cryab*-R120G-het vs. WT** | ***Cryab*-R120G-homo vs. WT** | **Retention Time (min)** | **Mass (Da)** |
| Cholest-7-en-3-ol (3β,5α) TMS | 834 | 6.14E-05 | -1.59 | -1.74 | -2.77 | 26.06 | 458 |
| unknown |  | 4.95E-08 | -1.17 | -1.89 | -2.21 | 12.02 | 73 |
| Benzene, (1-methyldodecyl) | 881 | 1.21E-04 | -2.66 | 1.38 | -1.93 | 15.46 | 105 |
| unknown |  | 2.30E-05 | -1.94 | 1.30 | -1.49 | 11.75 | 73 |
| D-(-)-Fructopyranose, 5TMS (isomer 1) | 904 | 4.60E-13 | -2.20 | 1.53 | -1.44 | 14.48 | 204 |
| unknown |  | 7.24E-16 | -2.15 | 1.72 | -1.25 | 10.42 | 71 |
| Arabinofuranose, 1,2,3,5-tetrakis-O-(TMS) | 676 | 0.010272 | 3.52 | -1.92 | 1.84 | 15.34 | 217 |
| L-Alanine, 2TMS derivative | 905 | 4.67E-12 | 1.06 | 1.75 | 1.85 | 5.32 | 116 |
| 9-Octadecenamide, (Z)- | 873 | 1.50E-05 | 1.74 | 1.08 | 1.88 | 19.55 | 72 |
| DL-Phenylalanine, TMS derivative | 864 | 6.14E-05 | 1.78 | 1.46 | 2.61 | 11.40 | 120 |
| L-Isoleucine, TMS derivative | 724 | 1.87E-06 | 2.87 | -1.07 | 2.69 | 6.43 | 86 |
| L-Valine, 2TMS derivative | 898 | 1.10E-15 | 1.59 | 1.72 | 2.74 | 6.98 | 144 |
| L-Leucine, TMS derivative | 845 | 1.22E-06 | 3.34 | -1.11 | 3.02 | 6.12 | 86 |
| L-Valine, TMS derivative | 843 | 2.31E-05 | 3.13 | -1.03 | 3.03 | 5.12 | 72 |
| L-Isoleucine, 2TMS derivative | 821 | 1.35E-11 | 1.87 | 1.78 | 3.33 | 8.09 | 73 |
| Lanosterol, TMS derivative | 729 | 3.39E-06 | 6.00 | -1.54 | 3.90 | 27.41 | 69 |
